# Molecular Diagnostic Biomarkers in Autism Spectrum Disorder: From Multi-Omics Signatures to Precision Therapeutic Targets

**DOI:** 10.3390/molecules31213952

**Published:** 2026-11-02

**Authors:** Yanghao Zheng, Mingzhe Zhao

**Affiliations:** Affiliated Mental Health Centre &Hangzhou Seventh People’s Hospital, Zhejiang University School of Medicine, Hangzhou 310013, China; zhengyanghao@zju.edu.cn

**Keywords:** autism spectrum disorder, molecular diagnostics, multi-omics, precision medicine

## Abstract

Autism spectrum disorder (ASD) is a systemic multi-organ condition characterized by profound biological heterogeneity. Clinical management currently faces a bottleneck due to reliance on subjective behavioral phenotyping rather than objective molecular markers. This review synthesizes breakthroughs in ASD diagnostics, moving from a reductionist “one-size-fits-all” approach toward an integrative systems biology framework. We evaluate how genetic variants and epigenetic modifications converge on shared pathways, such as synaptic homeostasis and chromatin remodeling. Furthermore, we highlight the diagnostic potential of proteomic and metabolomic signatures, which capture functional states more proximally to the clinical phenotype, including sex-specific metabotypes. The roles of neuroinflammation, oxidative stress, and the gut–microbiota–brain axis are delineated as key contributing factors of pathophysiology. Finally, we propose a roadmap for integrating multi-omics data via machine learning to facilitate early screening and precision therapeutic stratification. By bridging molecular footprints with clinical phenotyping, this synthesis provides a foundation for personalized medicine in neurodevelopmental disorders.

## 1. Introduction

Autism spectrum disorder (ASD) represents a cluster of heterogeneous neurodevelopmental condition defined by persistent deficits in social communication alongside restricted, repetitive patterns of behaviors [1]. Beyond these core neurobehavioral symptoms, ASD is increasingly conceptualized as a systemic multi-organ disorder. Clinical evidence suggests that the pathophysiology of ASD extends far beyond the central nervous system, frequently involving complex immunological dysregulation, gastrointestinal disturbances, and metabolic imbalances, all of which contribute to a staggering global socioeconomic burden [2,3].

The prevalence of ASD was estimated at 1 in 31 children in the United States and approximately 1 in 143 in China [4,5], attracting widespread societal attention. Currently, the “gold standard” for ASD diagnosis remains rooted in behavioral observations and caregiver reports, such as the Modified Checklist for Autism in Toddlers (M-CHAT), and Ages and Stages Questionnaire (ASQ) as screening tools, Autism Diagnostic Interview-Revised (ADI-R) and Autism Diagnostic Observation Schedule (ADOS) as diagnostic instruments, and Children Autism Rating Scale (CARS) as severity assessment tool [6]. While these psychometric instruments are indispensable and will remain foundational to clinical evaluation, they are inherently subjective and often fail to detect symptoms during the pre-symptomatic developmental period. Rather than serving as replacements for these established behavioral frameworks, emerging molecular biomarkers are intended to complement them by providing earlier pre-symptomatic risk stratification, objective biological subtyping, and real-time monitoring of therapeutic responses. This developmental window, typically before the age of two, represents a “golden period” of peak neural plasticity where intensive behavioral intervention has the maximum potential to normalize neurodevelopmental trajectories [7,8].

The profound biological heterogeneity of ASD, often referred to as “the Autisms”, suggests that a unified behavioral diagnosis masks a multitude of distinct molecular etiologies [9]. Therefore, it is critical to identify presymptomatic biomarkers that could complement the comprehensive clinical assessment of ASD and enable early intervention. The emergence of advanced molecular diagnostic approaches, including high-throughput Next-Generation Sequencing (NGS), single-cell transcriptomics, and deep-phenotyping metabolomics, has begun to unravel these convergent and divergent pathways [10].

In this review, we synthesize recent breakthroughs in the search for ASD biomarkers across genetic, epigenetic, proteomic, metabolic, immune, and microbiome domains. We critically evaluate how these molecular footprints can facilitate early screening and, crucially, act as therapeutic anchors for precision intervention. By bridging the gap between molecular diagnostics and clinical phenotyping, we aim to provide a strategic roadmap for the transition toward personalized medicine in neurodevelopmental disorders.

## 2. Methods

This article was prepared as a narrative review. The relevant literature was identified through searches of Pubmed (MEDLINE), Scopus, IEEE Xplore, Web of Sciences, and Google Scholar up to August 2026. Search terms included combinations of “autism spectrum disorder”, “genetic”, “epigenetic”, “Non-coding RNA”, “microRNAs”, “proteomic”, “metabolomic”, “immune dysregulation”, “neuroinflammation”, “oxidative stress”, “redox”, and “gut microbiota”. Additional articles were identified through citation tracking of relevant publications. Priority was given to peer-reviewed studies, clinical trials, and recent reviews relevant to molecular diagnostic biomarkers in ASD.

## 3. The Genetic and Epigenetic Landscape: From Genomic Architecture to Environmental Programming

**Figure 1:**
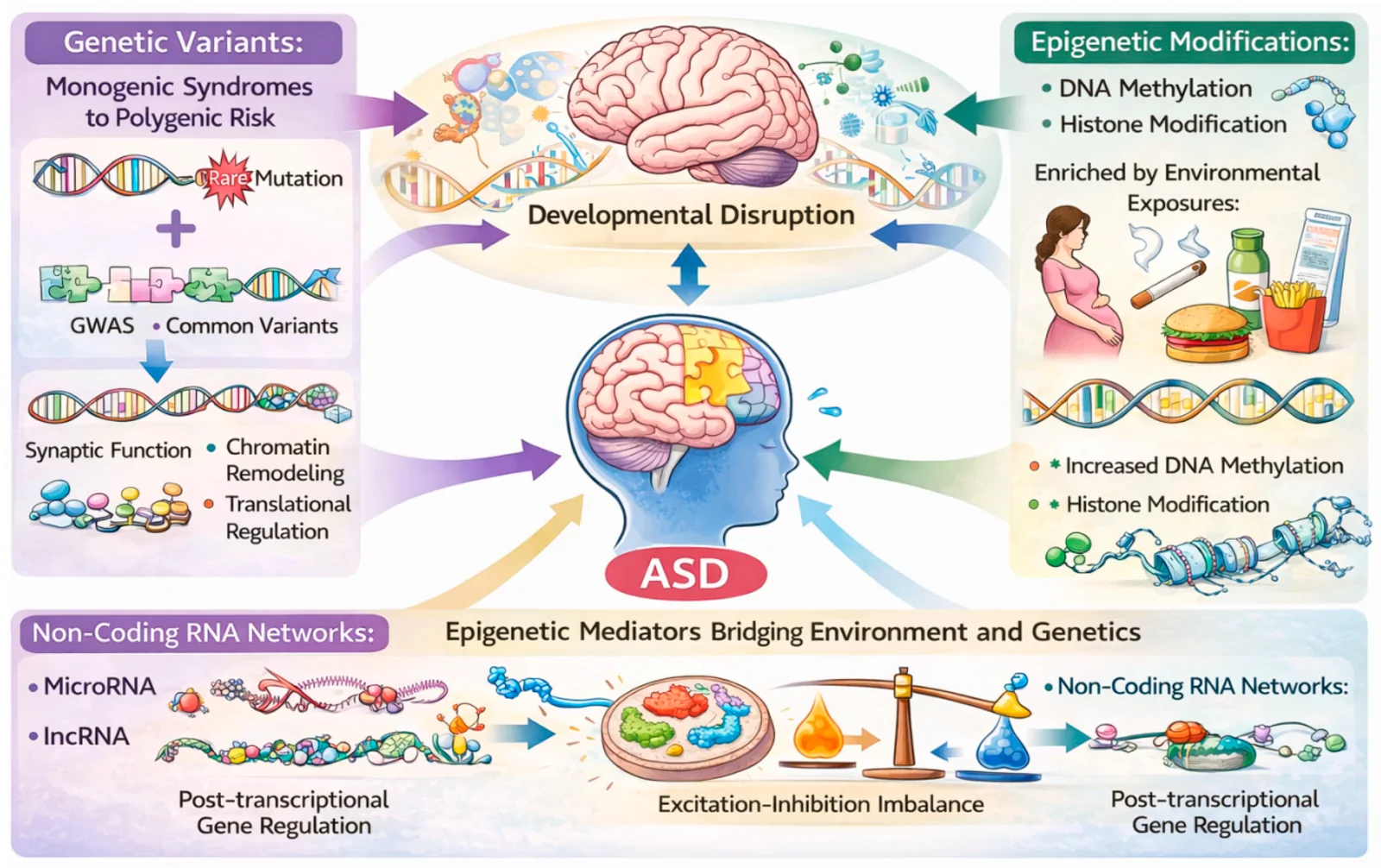
**The Multidimensional Molecular Landscape of ASD: From Genomic Architecture to Post-Transcriptional Regulation.** This schematic illustrates the hierarchical interplay between diverse molecular layers contributing to the pathophysiology of autism spectrum disorder (ASD). The Genomic Architecture layer represents the spectrum of genetic risk, ranging from rare, highly penetrant syndromic loci to common polygenic variants, which frequently converge on shared pathways like synaptic function and chromatin remodeling to drive excitatory–inhibitory (E/I) imbalance. Intersecting with this is the Epigenetic Mediators layer, where DNA methylation and histone modifications serve as “molecular memories” that integrate prenatal environmental exposures, such as maternal metabolic stress or hormonal influences, into lasting neurodevelopmental shifts, often leading to the silencing of master regulator genes. Finally, the non-coding RNA networks operate as molecular “rheostats,” where microRNAs (miRNAs) and long non-coding RNAs (lncRNAs) fine-tune gene expression post-transcriptionally. These molecules not only serve as dynamic biomarkers for liquid biopsy and symptom monitoring but also offer specialized avenues for RNA-based precision therapeutics to rescue protein haploinsufficiency. Collectively, these integrated molecular signatures transform ASD from a behavioral diagnosis into a molecularly stratified disorder. Note: The schematic illustrations in this figure were created with the assistance of AI tools and edited by the authors.

ASD arises from a complex interplay between inherited or de novo genetic variation and dynamic epigenetic regulation. While high heritability estimates, often exceeding 80%, highlight a substantial genetic contribution to ASD susceptibility [11,12], the pronounced phenotypic heterogeneity observed across individuals suggests that genetic architecture alone cannot fully explain disease manifestation. Epigenetic mechanisms, which integrate environmental exposures with genomic regulation, have therefore emerged as critical modulators of neurodevelopmental trajectories [13,14]. Importantly, both genomic variants and epigenetic signatures are increasingly recognized as potential molecular diagnostic markers capable of identifying biologically distinct ASD subtypes (Figure 1).

### 3.1. Genomic Landscapes: From Monogenic Syndromes to Convergent Pathways

The genetic architecture of ASD encompasses a wide spectrum of variation, ranging from rare highly penetrant de novo mutations to the cumulative effects of common polygenic risk variants [15,16]. Early insights into ASD genetics were largely derived from syndromic forms associated with single-gene mutations, including *FMR1* (Fragile X syndrome), *TSC1*/*TSC2* (tuberous sclerosis complex), *MECP2* (Rett syndrome), and *SHANK3* (Phelan-McDermid syndrome) [17,18,19,20]. However, large-scale sequencing studies, particularly whole-exome and whole-genome sequencing, have substantially expanded the catalog of ASD-associated loci. These efforts have revealed that diverse genetic variants frequently converge on shared neurodevelopmental pathways, including synaptic function, chromatin remodeling, and translational regulation.

More than 1200 genes have been implicated in ASD risk according to the SFARI Gene database, yet these loci rarely operate in isolation. Instead, integrative transcriptomic analyses demonstrate that many ASD-associated genes cluster within convergent functional modules governing neuronal differentiation, synaptic development, and chromatin regulation [21,22,23,24,25,26]. Single-cell transcriptomic studies further indicate that mutations in genes such as *SUV420H1*, *ARID1B*, and *CHD8* disproportionately affect specific neuronal populations during cortical development. These convergent disruptions are widely hypothesized to contribute to alterations in the balance between excitatory and inhibitory neural signaling, a mechanistic framework frequently referred to as the excitation–inhibition (E/I) imbalance model of ASD [24].

Importantly, emerging evidence suggests that therapeutic responsiveness may depend strongly on the underlying genetic context. For example, dietary zinc supplementation has been shown to rescue synaptic deficits in certain ASD-related genetic models, including Shank3-deficient mice, while similar interventions show limited effects in other genetic backgrounds such as Shank2 deficiency [27,28,29]. These findings indicated that Shank3 or Shank2 deficiency may serve as a predictive biomarker for the response to dietary zinc supplementation in ASD-related genetic models.

### 3.2. Epigenetic Mechanisms Linking Environmental Exposures to Gene Regulation

Epigenetic modifications, including DNA methylation and histone modifications, provide potential mechanisms through which prenatal environmental exposures may influence gene regulation and neurodevelopment. Maternal metabolic disorders, particularly pregestational and gestational diabetes, have been associated with an increased risk of ASD in offspring [30]. Epigenetic alterations have been proposed as one possible link between maternal metabolic disturbances and neurodevelopmental outcomes. For example, altered DNA methylation at the OR2L13 promoter has been implicated in ASD-related molecular changes, although its functional consequences for early neurogenesis require further investigation [31].

The “Extreme Male Brain” (EMB) theory has also prompted investigations into the contribution of prenatal hormonal exposure to ASD susceptibility [32]. Some studies have linked fetal steroid hormone levels to epigenetic regulation of RORA, including increased H3K9 trimethylation at its promoter and reduced transcriptional activity, although the evidence remains limited [33,34]. The explanatory scope of the EMB theory is also debated, as sex differences in ASD may reflect a combination of biological factors, diagnostic disparities, and behavioral camouflaging. Prenatal hormonal and epigenetic mechanisms may therefore contribute to ASD susceptibility in particular subgroups, but their role in explaining the male-biased prevalence of ASD remains unresolved.

### 3.3. Non-Coding RNA Networks: The Rheostats of Neurodevelopment

Beyond genetic and epigenetic variation, non-coding RNAs (ncRNAs), including microRNAs (miRNAs) and long non-coding RNAs (lncRNAs), represent an additional regulatory layer that modulates gene expression at the post-transcriptional level. These molecules fine-tune protein translation and cellular signaling pathways during neurodevelopment and in response to environmental stressors. While lncRNAs such as *NEAT1*, *TUG1*, and *AQP4-AS1* have demonstrated robust potential as peripheral diagnostic signatures [35,36], their functional significance is increasingly centered on their role as molecular effectors. For instance, the natural antisense lncRNA *RAB1B-AS1* interacts with the high-risk gene *CHD8* to modulate vesicle trafficking pathways, highlighting the intricate lncRNA-chromatin crosstalk inherent in ASD pathology [37].This mechanistic insight has paved the way for “RNA-based precision therapy,” exemplified by the development of synthetic SINEUPs-engineered ncRNAs designed to specifically boost endogenous protein levels, such as CHD8, to rescue haploinsufficiency-driven phenotypes [38].

Simultaneously, the inherent stability of miRNAs in various biofluids (serum, saliva, and feces) renders them promising candidate markers for non-invasive liquid biopsy [39]. However, the diagnostic utility of miRNAs is intrinsically linked to their highly dynamic expression profiles; evidence of opposing age-dependent correlations in toddlers versus older children underscores the necessity of considering temporal windows in biomarker validation (Table 1). Furthermore, the downregulation of *miR-484* not only facilitates the differentiation of ASD cohorts but also exhibits a significant inverse correlation with symptom severity [40]. This observation raises the possibility that miRNAs could act as a “molecular gauge”, offering a promising exploratory avenue for early screening research as well as real-time monitoring of disease progression and therapeutic response. These markers, however, remain candidate biomarkers and require extensive independent validation before clinical translation, and correlation does not confirm causal links between miRNA expression and ASD behavioral features.

Collectively, advances in genomic sequencing, epigenetic profiling, and RNA-based biomarker discovery hold potential to gradually shift ASD research beyond a purely behavioral diagnostic framework toward a model of molecular stratification. These insights provide a conceptual foundation for exploring the future integration of multi-omics candidate biomarkers into potential diagnostic frameworks, although such clinical implementation remains preliminary and unvalidated.

## 4. Targeted Proteomics and Metabolomics Diagnostic Markers

Proteomic and metabolomic profiling have emerged as central components of molecular diagnostic research in ASD, as they capture functional biological states that lie downstream of genetic and epigenetic regulation [66,67]. Unlike genomic variants, which primarily reflect susceptibility, protein and metabolite signatures provide dynamic readouts of cellular activity and physiological adaptation. Advances in high-resolution mass spectrometry and biofluid-based omics technologies have therefore enabled the identification of candidate molecular signatures detectable in peripheral samples, offering promising avenues for minimally invasive diagnostic development [68,69].

### 4.1. Proteomic Signatures: From Enzymes to Neural Homeostasis

Proteomic studies of ASD consistently identify alterations across several interconnected biological domains, particularly synaptic signaling [70], immune regulation [71], and cellular metabolism [72]. Early investigations relying on brain tissue analyses revealed dysregulation of synaptic scaffolding and neurotransmission-related proteins [73]; however, recent research has increasingly focused on peripheral biofluids, including plasma and serum, to identify clinically accessible biomarkers [67]. These studies report reproducible alterations in inflammatory mediators, complement cascade components, and oxidative stress-related proteins, suggesting systemic biological involvement beyond the central nervous system.

Importantly, only a subset of reported proteomic alterations demonstrates characteristics required for diagnostic translation. Several studies employing machine-learning-assisted proteomic panels have achieved moderate classification accuracy when distinguishing ASD from typically developing controls, highlighting the potential of multi-protein signatures rather than single biomarkers [68]. Nevertheless, variability across cohorts remains substantial, reflecting differences in age, comorbidities, analytical platforms, and sample processing protocols. Standardization and independent validation therefore represent critical prerequisites for clinical implementation.

### 4.2. Metabolomics and the “Sex-Bias” Paradigm

Metabolomics provides a direct snapshot of biochemical activity and has therefore become a particularly promising strategy for ASD molecular diagnostics [74]. Because metabolites integrate genetic predisposition, environmental exposure, diet, microbiome activity, and immune signaling, metabolic profiles may capture disease-relevant biology more proximally to phenotype than genomic markers alone. A particularly compelling frontier in metabolomics is the elucidation of sex-specific molecular signatures, which may explain the long-observed male bias in ASD prevalence [32]. Longitudinal analyses have revealed that female offspring with ASD often exhibit a disruption in the ether/non-ether phospholipid balance within maternal mid-gestation plasma [69]. In contrast, ASD-affected males tend to show a higher density of dysregulated chemical clusters in cord blood, suggesting that the “biological hits” leading to ASD may manifest through different metabolic pathways depending on biological sex [69]. This divergence supports the Female Protective Model, suggesting that different metabolic thresholds or compensatory mechanisms may exist across sexes, which is a critical consideration for developing sex-stratified diagnostic tools.

### 4.3. The Shift Toward Integrated Multi-Biomarker Panels

While individual biomarkers often exhibit variability across disparate cohorts due to the inherent heterogeneity of ASD, the field is rapidly pivoting toward multi-biomarker panels. Integrated models that synthesize multiple protein and metabolite signatures have consistently demonstrated superior diagnostic efficacy, often reaching high AUC values, compared to single-molecule approaches [67,75,76,77]. This transition from isolated markers to integrated metabolic profiles is essential for capturing the multifaceted nature of ASD. Moreover, these panels facilitate the identification of biologically meaningful metabotypes, paving the way for personalized metabolic interventions, such as targeted nutritional supplementation [78] or enzyme replacement therapies [79], tailored to the individual’s specific biochemical deficit. Looking forward, establishing robust and clinically actionable molecular diagnostic models will fundamentally rely on integrating these multi-biomarker frameworks with standardized clinical phenotyping and rigorous machine learning-based validation methodologies.

## 5. Immune Dysregulation and Neuroinflammation: Systemic Signatures and CNS Crosstalk

Accumulating evidence underscores that immune dysfunction is not merely a comorbid feature but play an important role in the pathophysiology of ASD [80]. This neuroinflammatory state is characterized by a persistent, low-grade systemic imbalance in the cytokine network, reflecting a broader immunological “priming”. Such priming suggests that the immune system of an individual with ASD is in a state of chronic readiness, where minor triggers can elicit exaggerated inflammatory responses that correlate with behavioral severity [81,82,83].

### 5.1. Systemic Cytokine Profiles: Molecular Correlates of Behavior

A growing body of evidence suggests that immune dysregulation contributes to ASD pathophysiology. Both central and peripheral immune alterations have been reported, including microglial activation within the developing brain and abnormal cytokine profiles in peripheral blood. Elevated levels of pro-inflammatory mediators such as interleukin-6 (IL-6), tumor necrosis factor-α (TNF-α), and interleukin-1β (IL-1β) have been detected in several independent cohorts of ASD individuals, suggesting persistent low-grade inflammatory activity [81,83].

Crucially, these molecular alterations are not mere epiphenomena; they serve as functional correlates of clinical symptoms. Specifically, elevated levels of IL-8 and MCP-1 (CCL2), chemokines involved in leukocyte trafficking, have been linked to the severity of social communication deficits and impaired cognitive flexibility. In contrast, high concentrations of TNF-α and IL-6 are frequently observed in subsets of patients exhibiting prominent irritability, stereotypic behaviors, and aggression [84,85]. This suggests that specific cytokine profiles could eventually serve as “biotypes” to predict behavioral trajectories and tailor behavioral interventions.

### 5.2. The IL-38/IL-36R Axis: A Failure of Homeostatic Buffering

A significant advance in understanding the systemic-CNS inflammatory dialogue is the discovery of the IL-38/IL-36R signaling axis. IL-38, an anti-inflammatory antagonist of the IL-1 family, represents a candidate biomarker for both diagnostic screening and therapeutic monitoring, though this potential remains preliminary and requires independent replication. A striking paradox has been observed: while children with ASD exhibit compensatory increases in serum IL-38, there is a substantial deficit of IL-38 and its cognate receptor, IL-36R, within the amygdala, a critical neural hub for emotional processing and fear responses [86].

This localized impairment in anti-inflammatory signaling within the brain suggests a “failure of homeostasis,” where the CNS is unable to utilize systemic anti-inflammatory signals to curb local neuroinflammation. Furthermore, the integration of advanced molecular imaging, such as PET tracers for the 18 kDa translocator protein (TSPO) to visualize microglial activation, provides an in vivo methodology to validate these findings. This non-invasive “liquid-to-imaging” correlation offers a robust biological gauge for assessing neuroinflammatory status and monitoring the brain’s response to systemic immunomodulatory therapies [87].

### 5.3. Immunomodulatory Paradigms: From “Dampening” to Precision

The recognition of neuroinflammation as a targetable driver has catalyzed investigations into various immunomodulatory interventions. Clinical trials have found that pharmacological agents, including celecoxib (a selective COX-2 inhibitor) and prednisolone, as well as nutritional strategies like omega-3 fatty acid supplementation, have shown preliminary efficacy in attenuating irritability and hyperactivity [88,89,90,91].

The therapeutic mechanism is increasingly thought to involve the reprogramming of microglial phenotypes from a pro-inflammatory (M1-like) to a neuroprotective (M2-like) state, alongside the restoration of the T-cell (Treg/Th17) balance [92]. However, the field must move beyond “dampening” general inflammation. The future of ASD immunotherapy lies in identifying individuals with specific immune biotypes who are most likely to respond to targeted cytokine inhibitors, thereby truly reshaping the neurodevelopmental trajectory through precision immuno-psychiatry.

Collectively, immune and microbiome research highlights the importance of systemic biological processes in ASD beyond the central nervous system. Although immune mediators and microbiome-derived metabolites show promise as peripheral biomarkers, current findings remain heterogeneous and require validation in larger, well-characterized cohorts. Integrating immune, microbiome, and multi-omics data may ultimately provide more robust molecular diagnostic signatures for ASD.

## 6. Oxidative Stress and Redox Imbalance: Molecular Fingerprints of Cytotoxicity

**Figure 2:**
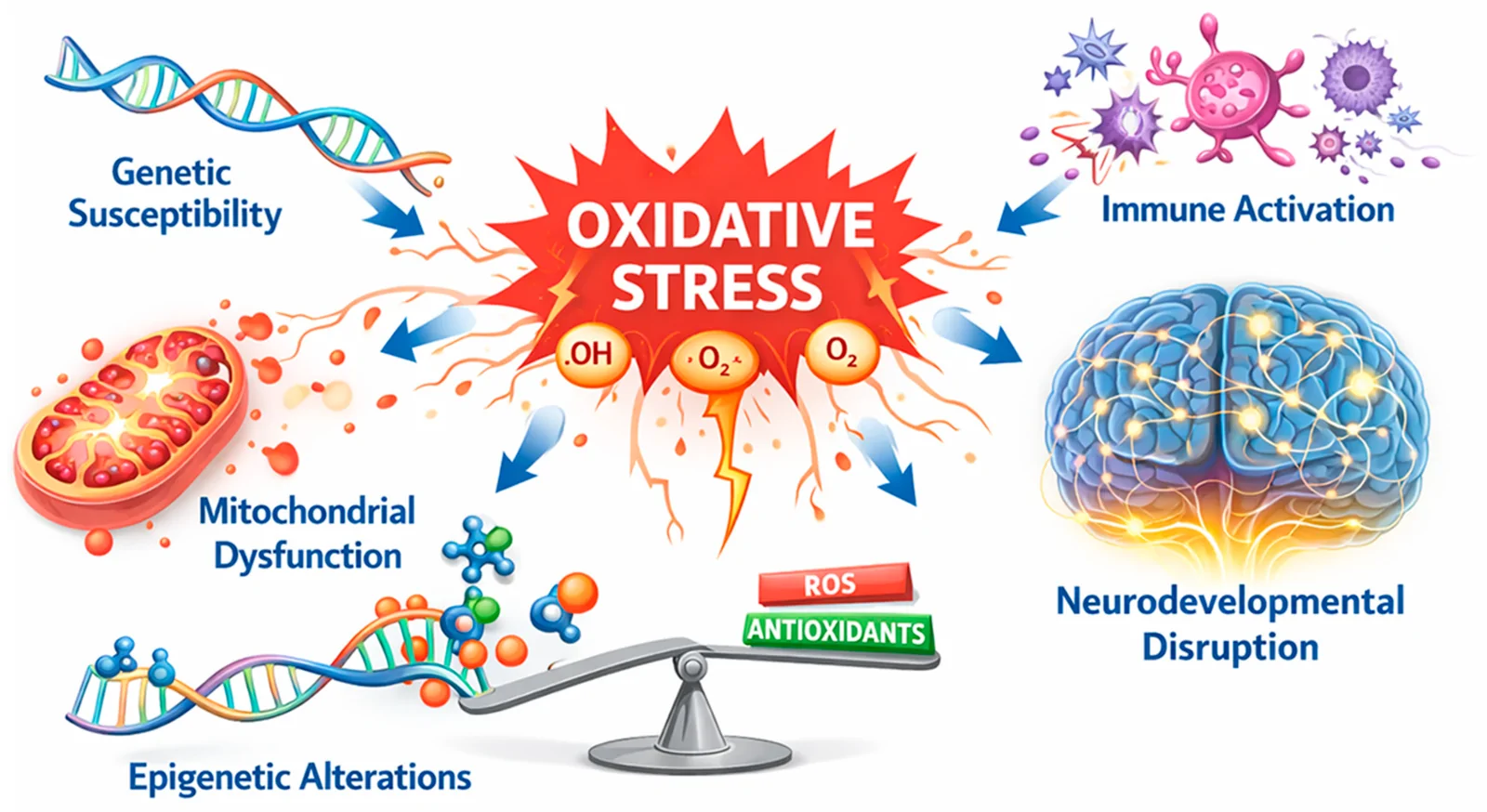
**Oxidative Stress as a Mechanistic Convergence Point in ASD Pathophysiology.** This schematic delineates the central role of redox imbalance in bridging diverse etiological factors with neurodevelopmental impairment in ASD. The diagram illustrates how genetic susceptibility, mitochondrial dysfunction, and environmental stressors (shown as upstream drivers) collectively trigger an oxidative surge. This systemic imbalance, characterized by the overproduction of reactive oxygen species (ROS) and a compromised antioxidant capacity, acts as a “developmental amplifier.” It leads to a cascade of cellular consequences, including the destabilization of mitochondrial bioenergetics and the manifestation of cytotoxic signatures within the neural environment. These oxidative insults eventually converge to impair synaptic plasticity and neurogenesis, serving as a critical interface through which disparate biological signals translate into the shared neurobiological and behavioral phenotypes characteristic of ASD. Note: The schematic illustrations in this figure were created with the assistance of AI tools and edited by the authors.

A growing body of evidence suggests oxidative stress not merely as a downstream consequence of ASD pathology but as a important integrative mechanism linking genetic susceptibility, mitochondrial dysfunction, immune activation, and altered neurodevelopmental trajectories [93] (Figure 2). Oxidative stress, defined as an imbalance between reactive oxygen species (ROS) production and antioxidant defense capacity, exerts disproportionate effects on the developing brain due to its high metabolic demand and limited antioxidant reserves. Emerging studies suggest that redox dysregulation acts as a developmental amplifier, destabilizing mitochondrial bioenergetics, reshaping epigenetic regulation, and impairing synaptic plasticity during critical windows of neural maturation [94]. Consequently, oxidative stress represents a mechanistic convergence point through which diverse etiological pathways translate into shared neurobiological phenotypes in ASD [94,95].

### 6.1. Multidimensional Cascades of Oxidative and Nitrative Damage

The accumulation of oxidative insults in ASD is evidenced by a coordinated elevation of biomarkers across multiple molecular strata, reflecting a systemic failure to contain reactive species. These molecular signatures should not be interpreted as isolated biomarkers but rather as interconnected layers of a systemic redox cascade, reflecting progressive cellular injury from membrane instability to genomic vulnerability. Lipid peroxidation represents an early and pervasive manifestation of this damage; elevated concentrations of malondialdehyde (MDA) and 4-hydroxynonenal (4-HNE) in the blood and urine of individuals with ASD indicate compromised cell membrane integrity and altered permeability, which are critical for proper neuronal signaling [96,97]. Furthermore, the consistent upregulation of 8-iso-prostaglandin F2α (8-iso-PGF2α), the recognized gold standard for lipid oxidative stress, signals a widespread systemic burden [98].

This lipid-level disruption is compounded by protein oxidation and nitrative stress, as evidenced by increased levels of protein carbonyls and 3-nitrotyrosine (3-NT), particularly within the cerebellum [95,99,100]. Such nitrative stress is frequently driven by excessive nitric oxide (NO) and peroxynitrite, which act as potent inhibitors of the mitochondrial electron transport chain, thereby impairing ATP production and fueling a vicious cycle of mitochondrial dysfunction and further ROS generation [101]. Ultimately, this oxidative siege extends to the genome; the elevation of 8-hydroxy-2′-deoxyguanosine (8-OHdG) in brain tissue and biofluids serves as a definitive indicator of chronic DNA oxidation [102,103,104]. Notably, the robust positive correlation between 8-OHdG levels and ASD severity positions this marker as a potentially invaluable molecular gauge for clinical staging and prognostic assessment, bridging the gap between metabolic disturbance and behavioral phenotype [105].

### 6.2. Compromised Antioxidant Defense and Redox Homeostasis

The severity of oxidative damage in ASD is exacerbated by a concurrent failure of the antioxidant defense system. Glutathione (GSH), the primary radical scavenger in the CNS, is significantly depleted in postmortem ASD brain samples [102,106]. A reduced GSH/GSSG ratio not only indicates poor antioxidant capacity but also correlates with the degree of language impairment and social deficits [107]. Notably, impaired antioxidant capacity in ASD appears to arise not solely from environmental exposure but from intrinsic metabolic vulnerability, including mitochondrial inefficiency and disrupted one-carbon metabolism. This suggests that redox imbalance represents a biologically embedded trait rather than a transient physiological state.

Furthermore, dysregulations in the methionine–homocysteine cycle, evidenced by reduced levels of vitamins B9, B12, and Sadenosylmethionine (SAM), suggest a systemic deficit in methylation capacity and thiol-based redox buffering [96]. While some studies report compensatory increases in erythrocyte superoxide dismutase (SOD) and catalase, the general trend toward reduced serum antioxidant levels reflects a tissue-specific failure to maintain redox homeostasis, leaving the developing brain susceptible to oxidative-induced neurotoxicity [108,109].

## 7. The Gut–Microbiota–Brain Axis: A Microbial–Metabolic Signaling Hub

**Figure 3:**
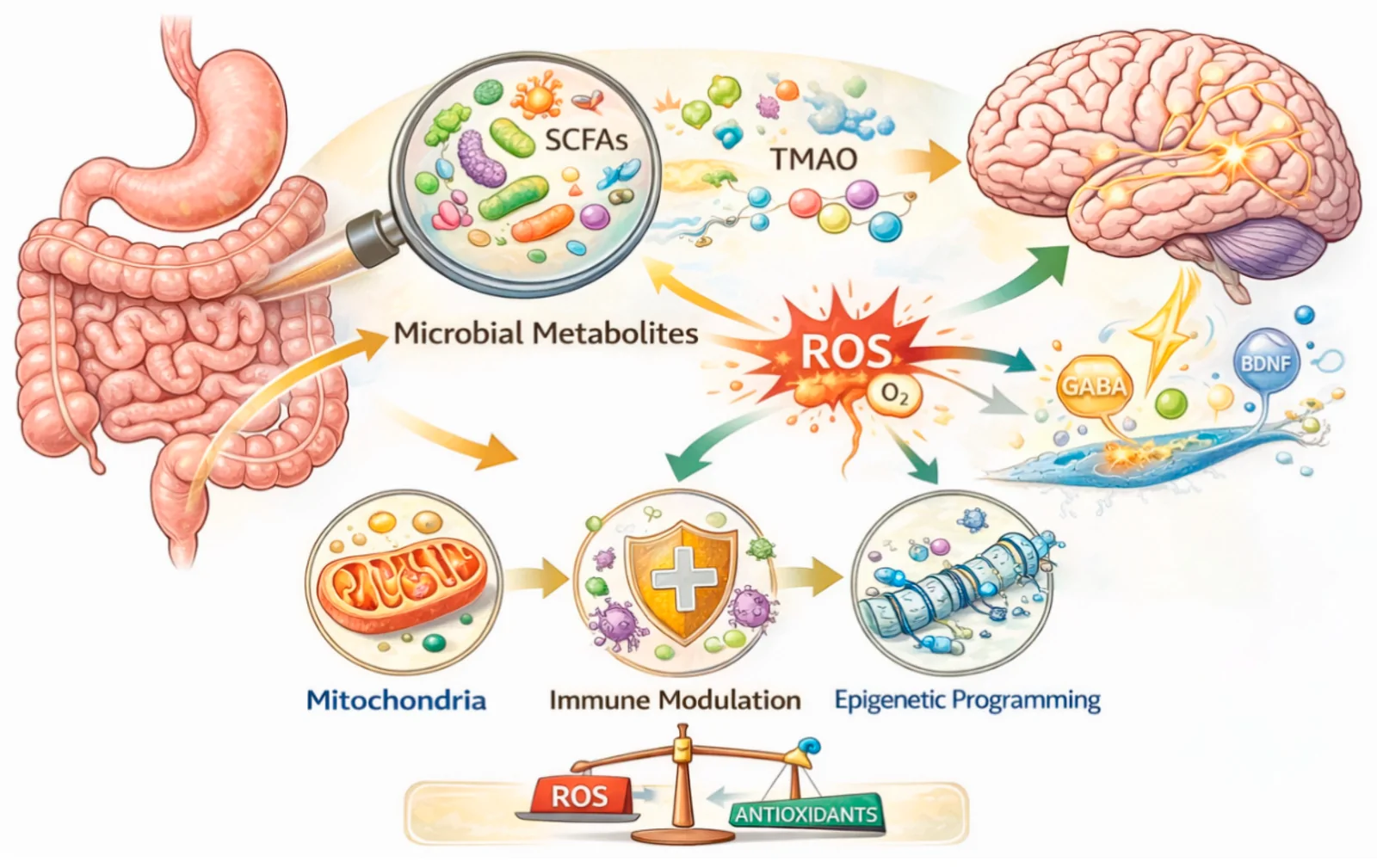
**The Gut**–**Microbiota**–**Brain Axis: A Microbial**–**Metabolic Signaling Network in ASD.** This schematic illustrates the systemic influence of the gut–microbiota–brain axis on neurodevelopmental trajectories in ASD. The diagram depicts how gut microbiota and their associated microbial metabolites, such as short-chain fatty acids (SCFAs) and trimethylamine N-oxide (TMAO), act as primary signaling molecules that bridge the gastrointestinal environment with the central nervous system. These metabolites exert their effects through multiple interconnected pathways, including the modulation of mitochondrial bioenergetics, immune system activation, and epigenetic programming. A central feature of this axis is the regulation of redox homeostasis; an imbalance between reactive oxygen species (ROS) and antioxidant defenses serves as a critical driver of neurodevelopmental disruption. Furthermore, microbial signaling influences the levels of key neurotransmitters and neurotrophic factors, such as GABA and BDNF, which are essential for synaptic maturation and cognitive function. Collectively, the gut-microbiota–brain axis functions as a biologically plausible interface where microbial dysbiosis translates into systemic metabolic and inflammatory signatures that converge on ASD vulnerability. Note: The schematic illustrations in this figure were created with the assistance of AI tools and edited by the authors.

Increasing evidence suggests that the gut–microbiota–brain axis represents a biologically plausible interface through which environmental exposures interact with host molecular systems during neurodevelopment [110]. Rather than acting solely through neural signaling pathways, gut microbiota exerts systemic effects via microbial metabolites, immune modulation, and redox regulation, thereby influencing mitochondrial function, epigenetic programming, and synaptic maturation (Figure 3). In ASD, microbial dysbiosis is increasingly interpreted not merely as a gastrointestinal comorbidity but as a potential regulator of metabolic and inflammatory pathways converging on neurodevelopmental vulnerability [111].

### 7.1. Microbial Dysbiosis and Targeted Metabolomics

Fecal metagenomic analyses consistently reveal significant alterations in the microbial landscape of children with ASD, typically characterized by an overgrowth of pathogenic taxa, such as *Clostridium* species, and a concurrent depletion of beneficial commensals including *Bifidobacterium* and *Bacteroidetes* [112,113] (Table 2). It is worth noting the marked inconsistency across studies, with some taxa reported as increased in ASD children in one investigation but decreased in another. Such discrepancies may stem from heterogeneity in participant geography, diet, age, gastrointestinal comorbidities, and prior medication exposure such as antibiotics or probiotics [114,115,116,117]. In addition, differences in stool handling, sequencing platforms, bioinformatic workflows and taxonomic resolution may contribute to divergent results. ASD heterogeneity itself may also lead to distinct microbial subtypes, which partially accounts for opposing abundance patterns [117].

However, taxonomic alterations alone provide limited mechanistic insight, as functional metabolic outputs, rather than microbial identity per se, are increasingly considered the principal mediators of microbiota–host neurobiological interactions. In this context, the diagnostic and mechanistic relevance of gut dysbiosis is increasingly attributed to microbial-derived metabolites that exert systemic biological effects.

Alterations in short-chain fatty acids (SCFAs), crucial regulators of blood–brain barrier (BBB) integrity, have been implicated in inducing behavioral deficits by modulating neuroinflammation [142,143,144]. In particular, SCFAs such as butyrate are capable of influencing host gene expression via epigenetic mechanisms, suggesting a potential molecular link between microbial metabolism and neurodevelopmental regulation. Furthermore, elevated levels of microbial metabolites including p-cresol and 4-ethylphenol in biofluids not only distinguish ASD cohorts but also correlate with symptom severity, supporting their potential utility as candidate metabolomic biomarkers reflecting gut ecosystem disruption [145,146]. Complementary evidence indicates dysregulation of bile acid and tryptophan metabolic pathways, highlighting a broader metabolic interface through which intestinal dysbiosis may contribute to systemic biochemical imbalance and altered neurodevelopmental signaling.

### 7.2. Intestinal Permeability and Microbial-Metabolic Crosstalk

Altered intestinal barrier integrity has frequently been reported in individuals with ASD, with increasing evidence suggesting that enhanced intestinal permeability, often referred to as a “leaky gut”, may contribute to systemic inflammatory signaling. Molecular profiling of duodenal biopsies has revealed downregulation of key barrier-forming tight junction proteins (e.g., CLDN1, OCLN) accompanied by increased expression of pore-forming claudins, a shift that may compromise epithelial integrity and facilitate translocation of microbial products [147,148]. These alterations have been partially attributed to reductions in butyrate-producing bacterial populations, which play a critical role in maintaining epithelial homeostasis and anti-inflammatory signaling within the intestinal mucosa.

Beyond bacterial compositional shifts alone, emerging research underscores that ASD-associated microbial alterations are intimately linked to functional metabolic and biochemical interfaces. Advanced metagenomic and metabolomic profiling have identified integrated biomarker panels comprising specific bacterial signatures and downstream metabolic products that demonstrate high discriminative performance for ASD across independent cohorts [149]. The reproducibility of these microbial–metabolic signatures across different populations suggests that functional gut ecosystem readouts may provide robust biological insights. Integrating such multi-layer microbial profiles with host-derived molecular markers may therefore represent a promising direction for developing robust and biologically informed diagnostic frameworks for ASD.

## 8. Methodological and Translational Hurdles from Multi-Omics Discovery to Clinical Implementation

A critical challenge underlying peripheral ASD biomarkers is their modest diagnostic specificity. Alterations in circulating cytokines, systemic redox states, mitochondrial metabolites, and gut microbiota profiles are non-specific pathophysiological signatures shared across various neurodevelopmental, psychiatric, and metabolic conditions, including schizophrenia, major depressive disorder, epilepsy, and inflammatory bowel diseases. Consequently, individual markers in these pathways often reflect broad biological stress or systemic dysregulation rather than ASD-exclusive etiology, underscoring why multi-domain biomarker integration and inclusion of non-ASD clinical controls are essential to isolate disease-specific profiles.

Translating multi-omics findings into clinically applicable diagnostic tools for ASD remains challenging. A critical conceptual distinction that must be emphasized is the boundary between a statistically significant molecular difference identified in group-level comparisons and a clinically useful diagnostic biomarker. While observational cohorts frequently report molecular features with statistically significant differences between ASD and control groups, statistical significance alone does not demonstrate clinical diagnostic utility. Clinical implementation additionally demands rigorous analytical validity, predefined decision thresholds, clinical validity, and demonstrated clinical utility. A major limitation is the high dimensionality of omics data relative to the sample sizes of most clinical cohorts, which increases the risk of overfitting and limits model generalizability [150,151]. Data leakage during feature selection or hyperparameter tuning can further inflate predictive performance. Nested cross-validation, prespecified preprocessing pipelines, and independent external validation are therefore essential for obtaining reliable performance estimates [151,152]. Technical heterogeneity introduces additional complexity. Differences in sample handling, laboratory protocols, and analytical platforms can produce batch effects that obscure disease-associated signals. Although harmonization methods can mitigate some of this variation, their suitability depends on the data type and study design. For example, ComBat-seq was developed for RNA-sequencing count data and is not directly applicable to all omics modalities [153]. Moreover, when diagnostic groups are confounded with processing batches, technical and biological effects may be difficult to disentangle. Appropriate quality control and validation across analytical settings are therefore necessary to establish the reproducibility of identified biomarkers.

Class imbalance and inadequate model calibration also complicate clinical translation. Unequal representation of ASD cases and neurotypical controls may bias classifiers toward the majority class, while resampling and cost-sensitive learning do not necessarily resolve all performance limitations [154]. Furthermore, high classification accuracy does not guarantee reliable individual-level predictions. Model evaluation should therefore consider discrimination, sensitivity, specificity, predictive values, and calibration, using measures such as the Brier score and calibration plots [155]. Performance must also be interpreted in relation to the target population, as predictive values and estimated risks may differ substantially between case–control studies and routine clinical settings. Generalizability across populations remains a further concern. Biomarker signatures derived from single-center or relatively homogeneous cohorts may reflect differences in ancestry, age, sex, developmental stage, or environmental exposure rather than ASD-related biology alone [156]. Given the clinical and etiological heterogeneity of ASD, validation in independent, geographically and ancestrally diverse cohorts is essential. Subgroup-specific performance should also be examined to identify potential disparities in diagnostic accuracy and determine whether model recalibration or updating is required [152].

Finally, clinical adoption depends on both interpretability and demonstrated utility. Explainable artificial intelligence methods, including SHapley Additive exPlanations (SHAP) and Local Interpretable Model-agnostic Explanations (LIME), can help characterize how individual molecular features contribute to model predictions [157]. However, feature attribution does not establish biological causality, and apparent associations may arise from confounding or correlated features. Integrating computational predictions with biological evidence and independent replication is therefore crucial. Ultimately, clinical translation will require reproducible performance, demonstrable incremental value over existing diagnostic assessments, and prospective evaluation in real-world settings [152,158].

## 9. Conclusions and Future Perspectives

The expanding body of evidence reviewed herein highlights that neurodevelopmental conditions such as ASD cannot be adequately explained by molecular perturbations within a solitary biological layer. Instead, ASD arises from dynamic, bidirectional interactions across genetic, epigenetic, metabolic, mitochondrial, immune, and microbial networks, which collectively shape atypical neurodevelopmental trajectories. This systems-level paradigm challenges traditional reductionist diagnostics and underscores the necessity of integrated molecular frameworks [Supplementary Table S1].

Genomic studies have successfully mapped numerous risk loci associated with neurodevelopmental vulnerability; however, their limited predictive power at the individual level demonstrates that genetic predisposition alone fails to capture full phenotypic heterogeneity. Epigenetic regulation serves as a critical interface through which environmental exposures, metabolic states, and cellular stress responses modulate gene expression during vulnerable developmental windows. In parallel, mitochondrial dysfunction and systemic redox imbalance have emerged as convergent pathophysiological nodes, bridging metabolic disturbances with aberrant neuronal signaling, impaired synaptic plasticity, and chronic neuroimmune activation. Furthermore, microbial and metabolic derivatives act as systemic modulators influencing blood–brain barrier permeability, neuroinflammation, and host metabolic homeostasis. The convergence of these interconnected pathways indicates that ASD pathophysiology is fundamentally driven by network-level dysregulation rather than isolated molecular anomalies. Consequently, single-biomarker diagnostic models are inherently inadequate for capturing this multidimensional biology.

**Figure 4:**
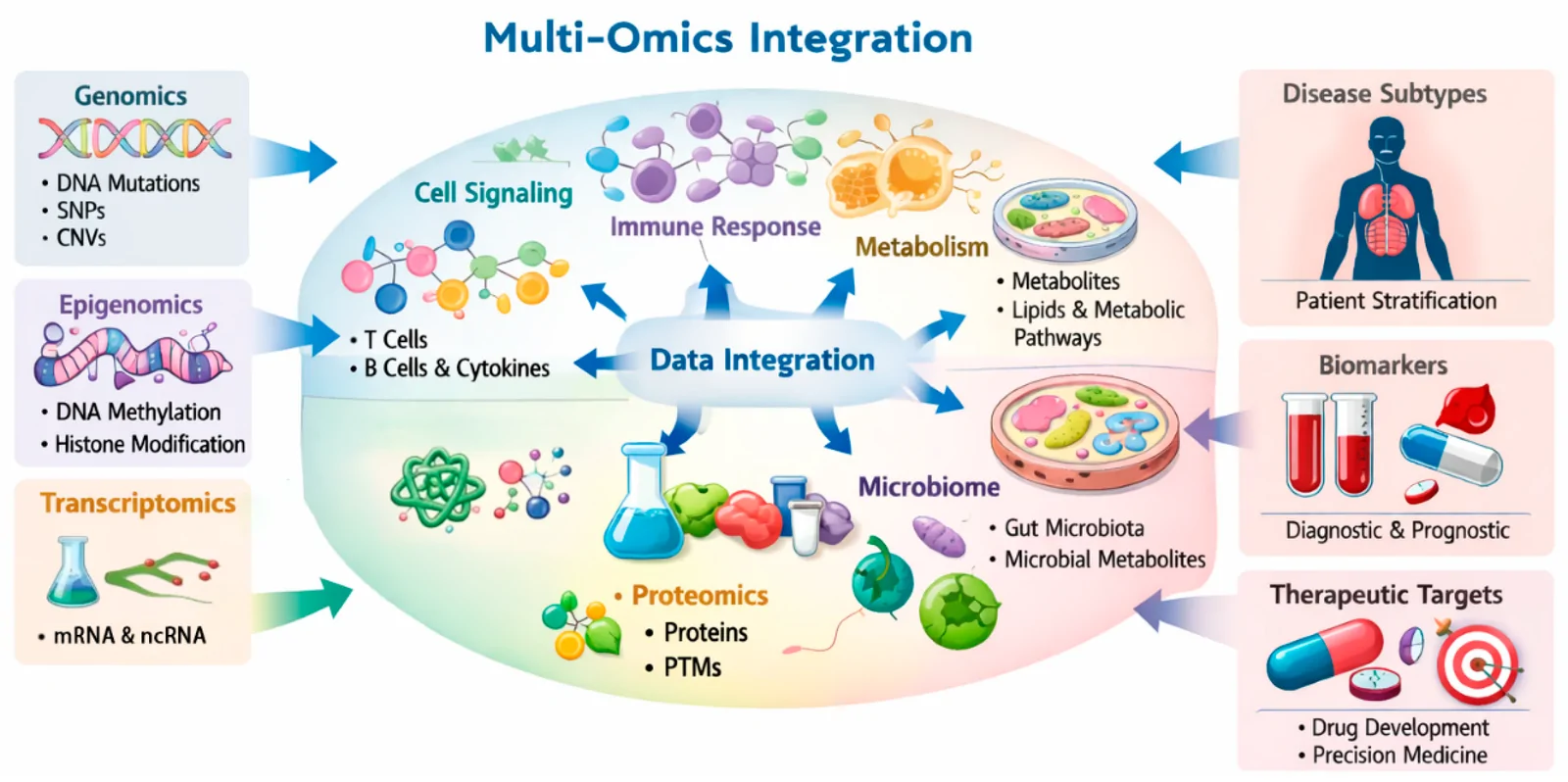
**Future Framework for ASD Precision Medicine: Multi-Omics Integration and Systems-Based Stratification**. This schematic presents a conceptual framework for the next generation of molecular diagnostics and personalized intervention in ASD. The framework initiates with the systematic acquisition of high-dimensional datasets (**left**), encompassing genomic susceptibility markers, epigenetic signatures, and non-coding RNA profiles, alongside functional readouts from proteomics. At the heart of this model is a centralized Data Integration hub (**center**), which serves to synthesize these heterogeneous biological streams, including immune signaling, metabolic pathways, and microbiome features, to decode the complex, interconnected networks underlying the disorder. This systems-level integration facilitates a multi-faceted clinical transition (**right**), moving beyond traditional behavioral assessments toward early biomarker detection, biologically driven patient stratification, and the development of personalized therapeutic targets and precision medicine strategies. Ultimately, this integrated approach establishes a robust molecular foundation for improving long-term clinical outcomes and treatment monitoring in ASD. Note: The schematic illustrations in this figure were created with the assistance of AI tools and edited by the authors.

Looking forward, clinical diagnostics must pivot toward multi-omics synthesis, combining genetic risk scores, epigenetic signatures, metabolic profiles, mitochondrial functional markers, and microbial signatures (Figure 4). Although advanced machine learning and network-based algorithms provide robust analytical tools to decipher these heterogeneous datasets, a realistic appraisal of translational readiness is essential. While peripheral immune profiles, select microbial metabolites, and specific metabolic ratios exhibit emerging consistency across independent cohorts, the vast majority of candidate biomarkers remain strictly in the exploratory discovery phase. They continue to be constrained by modest sample sizes, batch effects, cross-platform technical variance, and a paucity of longitudinal validation. Accordingly, integrated multi-omics diagnostics are not yet mature for routine clinical deployment.

In summary, shifting the paradigm from single-domain discovery to systems-driven biomarker panels holds profound promise for precision psychiatry. Realizing this clinical potential, however, will depend on rigorous, multi-center replication, standardized analytical pipelines, and longitudinal tracking, ultimately paving the way for objective, biology-informed stratification and early intervention in ASD.

## Figures and Tables

**Table 1:** **Alterations** **of miRNA expression in different biological samples of ASD patients.**

Biological Samples	Sample Population	Sample Size	Demographic Characteristics	Analysis Method	Upregulated miRNAs in ASD	Downregulated miRNAs in ASD	Main Findings	References
Whole blood	Children	7	5–15 years	RT-PCR	miR-34c-5p, -145-5p, -92a-2-5p, -199a-5p,-134-5p, -138-5p	miR-19b-1-5p, -27a-3p, -193a-5p	The main targets of these miRNAs are involved in immunological developmental, immune response and protein synthesis at transcriptional and translational levels;	[41]
			7.5 years Male: 7					[42]
	Adult	20	30.0 ± 8.2 years Male: 13	RT-PCR	miR-15b-5p	miR-6126	miR-15b-5p may regulate TGFBR3 mRNA expression and IQ was significantly associated with TGFBR3; miR-6126 is negative correlated with severity of the Social Response Scale in adults with high functioning ASD;	[43]
		30	28.4 years Male: 18					[44]
Blood plasma	Children	30	3–11 years Male: 26	RT-PCR	miR-365a-3p, -619-5p, -664a-3p, -197-5p, -424-5p	miR-3135a, -328-3p, -197-5p, -500a-5p, -424-5p, -484	The combination of miR-500a-5p and miR-197-5p achieves exceptional diagnostic accuracy for ASD, with a combined AUC of 0.975; miR-484 is negatively related to ASD severity;	[45]
		38	Male:30					[46]
		103	6.9 ± 3.0 years Male: 68					[40]
		45	2–13 years Male: 31	PCR		miR-3613-3p, -150-5p, -19a-3p, -361-5p, -499a-5p, -126-3p	miR-126-3p is a promising biomarker for early and accurate ASD diagnosis;	[47]
		4	6–20 years Male: 4	“silicon-on-insulator”-based nanosensor (SOI-NS)	miR-494-5p, -15b-5p, -106a-5p			[48]
		66	2–4 years Male: 54	High-throughput sequencing of miRNAs	miR-15b-3p, -30c-5p, -4433b-5p, -15a-5p, 335-5p, -3613-5p, -548ad-5p, -365a-3p	miR-532-5p, -1180-3p, -92b-3p, -92b-5p, -1299	miR-532-5p, miR-15b-3p, and miR-30c-5p are positively correlated with ASD severity; miR-4433b-5p, miR-15a-5p, miR-335-5p, and miR-1180-3p exhibit high diagnostic accuracy, with ROC-AUC of 0.936;	[49]
Blood serum	Children	105	11.3 ± 5.4 years Male: 88	miRNA sequencing	miR-206, -184, -223-5p, -4732-5p, -193b-5p	miR-576-3p, -193a-5p, -27a-5p, -379-5p, -134-5p, -574-3p, -382-5p, -7-5p, -103a-3p, -378a-3p, -3614-5p, -873-3p, -433-3p	miR-27A-5P, miR-382-5P, miR-223-5P, miR-103A-3P are associated with symptom severity;	[50]
		60	7.1± 2.6 years	qPCR	miR-21-5p, -101-3p, -106b-5p, -19b-3p, -195-5p, -130a-3p	miR-3613-3p, -150-5p, -126-3p, -361-5p, -19a-3p, -499a-5p, -151a-3p, -181b-5p, -320a, -328, -433, -489, -572, -663a	miR-21-5p shows a diagnostic ability in ASD patients (AUC = 0.921); miR-181b-5p, miR-320a, miR-572, miR-130a-3p, and miR-19b-3p demonstrate high diagnostic value with high sensitivity, specificity and AUC; miR-195-5p correlates positively with ASD severity, whereas miR-499a-5p correlates negatively;	[51]
		45	2-13 years Male: 31					[52]
		30	4.5 ± 1.8 years Male: 22					[53]
		40	4.0 ± 0.2 years Male: 33					[54]
		30	3–11 years Male: 24	RT-PCR		miR-3135a, -328-3p, -137	miR-3135a and miR-328-3p combination yields improve diagnostic accuracy (AUC 0.858);	[55]
Saliva	Children	187	2–6 years Male: 161	High-throughput sequencing of miRNAs	miR-665, -4705, -620, -1277-5p, -1246, -199b-5p, -4516, -199a-3p, -199b-3p, -10a, -16-5p	miR-28-3p, -148a-5p, -151a-3p, -125b-2-3p, -7706, -545-5p, -660-3p, -4284, -203a-5p, -1973, -451a, 146a-5p, -27a-3p,	miR-125b-2-3p and miR-148b-3p are associated with social affect; miR-451a is the most relevant to clinical practice and is associated with impaired social interaction;	[56]
		5	3-7 years Male: 4					[57]
		39	4–9 years Male: 25	qPCR	miR-7-5p, -2467-5p, -29a-3p, -141-3p	miR-23a-3p, -32-5p, -140-3p, -628-5p, -16-5p, -451a	miR-451a and miR-29a-3p are associated with anomalies of social interaction and communication;	[58]
		76	6.9 ± 1.5 years Male: 60					[59]
		24	9.2 ± 2.5 years Male: 16	RNA-Sequence	miR-7-5p, -28-5p, -127-3p, -140-3p, -191-5p, -218-5p, -335-3p, -628-5p, -2467-5p, -3529-3p	miR-23a-3p, -27a-3p, -30e-5p, -32-5p	miR-335-3p shows a diagnostic ability in ASD patients (AUC = 0.92); miR-30e-5p has the highest accuracy in predicting ASD diagnosis at 76%	[60]
Cerebellar cortex		242		RNA-Sequence	miR-10a-5p, -21-3p, -18b-5p, -20b-5p, -18b-5p, -20b-5p, -424-5p, -425-3p, -449b-5p, -450b-5p, -484, -629-5p, -651-5p, -708-5p, -766-3p, -874-3p, 887-3p, -940, -1277-3p, -3938, -2277-5p, -23a-3p, -107, -129-2-3p, -130b-5p, -148a-3p, -155-5p, -218-2-3p, -221-3p, -223-3p, -335-3p, -363-3p, -424-3p	can_1002-m, miR-204-3p, -491-5p, -619-5p, -3687, -5096	hsa-miR-21-3p targets neuronal genes down-regulated in ASD; hsa_can_1002-m regulates the epidermal growth factor receptor and fibroblast growth factor receptor signaling pathways involved in neural development and immune function;	[61]
Frontal cortex		12	18–51 years Male: 10	RT-PCR	miR-142-5p, -142-3p, -451a, -144-3p, -21-5p, -7-5p, -19a-3p, -19b-3p, -21-3p, -146a-5p, -155-5p, -219-5p, -338-5p, -379-5p, -451a, -494, -3168	miR-34a-5p, -92b-3p, -211-5p, -3960	hsa-miR-142 overexpression correlates with decreased methylation of its promoter region in the brain of individuals diagnosed with ASD; miR-21-5p and miR-451a target biological pathways and specific genes that are highly relevant to ASD;	[62]
Temporal gyrus	Children	5	4–9 years Male: 3	RNA-Sequence	miR-146a		miR-146a upregulation occurs early during childhood in the ASD brain.	[63]
Superior temporal sulcus and primary auditory cortex		10	31.0 ± 5.3 years Male: 5	RNA-Sequence	miR-664-3p, -4709-3p, -4753-5p, -1260b	miR-1, -297, -4742-3p, -93-3p, -424-3p, -484, -3607-5p, -3916	Dysregulated miRNAs in ASD are linked to nervous system function, cell cycle, and signaling pathways such as PI3K-Akt;	[64]
		8	5–50 years Male: 4					[65]

**Table 2:** **Summary** **of studies on gut microbiota alterations in children with ASD over the past 5 years.**

Taxonomic Level	Taxa	Gram Classification	Research Method	Abundance in ASD vs. Controls	References
Phylym	*Actinobacteria*	Positive	16S rRNA	↑	[118,119,120,121,122]
				↓	[123,124]
Phylym	*Bacteroidetes*	Negative	16S rRNA	↑	[125]
				↓	[119,120,126]
Phylym	*Firmicutes*	Positive	16S rRNA	↑	[119,124]
			metagenomic sequencing		[127]
			16S rRNA	↓	[121,125]
Phylym	*Firmicutes*/*Bacteroidetes ratio*	/	16S rRNA	↑	[119,120]
				↓	[125,128]
Phylym	*Proteobacteria*	/	16S rRNA	↑	[119,121,122,123]
			metagenomic sequencing		[127]
			16S rRNA	↓	[125]
Phylym	*Verrucomicrobia*	Negative	16S rRNA	↓	[125]
Class	*Bacilli*	Positive	16S rRNA	↑	[118]
Order	*Enterobacteriales*	Negative	16S rRNA	↑	[129]
Family	*Enterobacteriaceae*	Negative	16S rRNA	↑	[122,130,131]
				↓	[125]
Family	*Lachnospiraceae*	Positive	16S rRNA	↑	[129]
			metagenomic sequencing		[127]
			16S rRNA	↓	[122,126]
Genus	*Bacteroides*	Negative	16S rRNA	↑	[118,125,128,132]
			metagenomic sequencing		[127]
			16S rRNA	↓	[120,129]
			metagenomic sequencing		[133,134]
Genus	*Bifidobacterium*	Positive	16S rRNA	↑	[119,120,135,136]
				↓	[128,137]
			metagenomic sequencing		[127]
Genus	*Blautia*		16S rRNA	↑	[135,136]
Genus	*Clostridium*	Positive	16S rRNA	↑	[113]
			metagenomic sequencing		[138]
			16S rRNA	↓	[118]
Genus	*Collinsella*	Positive	16S rRNA	↑	[130,132]
				↓	[129]
Genus	*Coprococcus*	Positive	16S rRNA	↓	[132]
Genus	*Dialister*	Negative	16S rRNA	↑	[119]
			metagenomic sequencing		[138]
			16S rRNA	↓	[125]
			metagenomic sequencing		[133,134]
Genus	*Eggerthella*	Positive	16S rRNA	↑	[113]
Genus	*Enterococcus*	Positive	16S rRNA	↑	[113]
Genus	*Escherichia-Shigella*	Negative	16S rRNA	↑	[119,122,130,131,139]
				↓	[125]
Genus	*Faecalibacterium*	Positive	16S rRNA	↑	[124,132,136]
				↓	[129]
			metagenomic sequencing	↓	[138]
Genus	*Flavonifractor*	Positive	16S rRNA	↓	[125]
Genus	*Klebsiella*		16S rRNA	↑	[139]
Genus	*Lactobacillus*	Positive	16S rRNA	↑	[130]
Genus	*Nitriliruptor*	Positive	16S rRNA	↑	[140,141]
Genus	*Oscillospira*	Positive	16S rRNA	↑	[137]
Genus	*Phyllobacterium*	Negative	16S rRNA	↑	[130,131]
Genus	*Prevotella*	Negative	16S rRNA	↑	[124,125,139]
			16S rRNA	↓	[132]
			metagenomic sequencing		[127,134]
Genus	*Roseburia*	Positive	16S rRNA	↓	[130]
Genus	*Ruminococcus*	Positive	16S rRNA	↑	[124,128,137]
				↓	[118]
Genus	*Sutterella*	Negative	16S rRNA	↑	[132]

Note: ↑, increased abundance in ASD patients compared to healthy controls; ↓, decreased abundance in ASD patients compared to healthy controls.

## Data Availability

No new data were created or analyzed in this study.

## Supplementary Materials

The following supporting information can be downloaded at: https://www.mdpi.com/article/10.3390/molecules31213952/s1, Table S1: Summary of major candidate biomarkers for autism spectrum disorder.
