# Supplementary material for: Molecular Diagnostic Biomarkers in Autism Spectrum Disorder: From Multi-Omics Signatures to Precision Therapeutic Targets
Source: Molecules. 2026 Nov 2;31(21):3952. doi: 10.3390/molecules31213952 (PMC13764723; doi:10.3390/molecules31213952)
Supplement: Supplementary file 1 [file molecules-31-03952-s001.zip › molecules-4585836-supplementary.pdf]

Supplementary Table S1. Summary of major candidate biomarkers for autism spectrum disorder.

Biomarker/ biomarker panel	Biological samples	Sample population	Demographic characteristics	Sample size	Analytical platform	Performance metrics: AUC (95%CI), sensitivity, specificity	Proposed clinical application	Evidence types	Model validation strategy	References
Maternal diabetes	NA	Mother-infant pairs	Offspring of women with type 1 diabetes: type 2 diabetes: unexposed	29,094: 29,745: 3,186,305	Cox proportional hazard model	NR	Risk prediction	Cohort study	NA	[1]
Cortisol, estradiol and 17-hydroxyprogesterone	Serum	Mother in the first trimester of gestation, offspring	Offspring ASD with intellectual disability (ID): ASD without ID: control	118: 249:477	Restricted cubic spline logistic regression models	NR	Risk prediction	Case-control study	NA	[2]
Combined expression of *RORA*, *SOD2*, *GPER* and *PGR*	Umbilical cord blood mononuclear cell	Children with ASD	ASD: control<br>Male: 36<br>26.3 ± 1.6 months	41: 41	NA	AUC=0.87(standard deviation 3.9%)<br>sensitivity=1.000<br>specificity=0.854	Risk prediction	Longitudinal follow-up study	NA	[3]

20 significant ncRNAs	Peripheral blood	Children with ASD	ASD (male: 80, 8.1 years)<br>Control (male:48, 8 years)	104: 82	LASSO penalized generalized linear model	Training set: AUC=0.96<br>validation set: AUC=0.97<br>external validation set: AUC= 0.96	Candidate diagnostic marker	Case-control study	Cross-validation, external validation	[4]
a panel composed of 15 proteins	Serum	Children with ASD	ASD: control<br>6-15 years	91:30	Olink proximity extension assay	AUC=0.876（95%CI：0.697-1.000）<br>sensitivity=0.999（95%CI：0.997-1.000）<br>specificity=0.662（95%CI：0.338-0.986）	Candidate diagnostic marker	Case-control study<br>pilot discovery	four-fold cross-validation	[5]
a panel composed of 12 differentially-expressed proteins	plasma	Children with ASD	ASD : control<br>male: 24	30：30	Machine Learning Techniques	Training set: AUC=0.96, sensitivity= 1<br>validation set: AUC=0.97, sensitivity=0.88	Candidate diagnostic marker	Case-control study	Cross-validation	[6]

Age, gender and CML, CMA, DT, 3DG-H and AVE	plasma	Children with ASD	ASD (male: 247): Control (Male: 94) 5-12 years	311: 167	LC-MS/MS	Accuracy=0.89（95% CI 0.82-0.96）sensitivity=0.91（95% CI 0.82-0.99）specificity=0.74 (95% CI 0.68-0.80)	Candidate diagnostic marker	International multicenter clinical validation study	Independent multicenter external validation	[7]
IL-8	plasma	Children with ASD	ASD: Control	45: 38	Human Cytokine Twenty-Five -Plex Kit	AUC=0.858（95%CI：0.777-0.939）	Candidate diagnostic marker	Case-control study	NR	[8]
Oxidative stress markers	urine	Children with ASD	low functioning ASD: medium functioning autism: high functioning autism: control 4-12 years	15: 15: 15: 50 (male: female=4:1)	UV spectrophot-ometric	NR	Candidate marker	Case-control study	NR	[9]

1. Feig DS et al: Long-term Neurobehavioral and Metabolic Outcomes in Offspring of Mothers With Diabetes During Pregnancy: A Large, Population-Based Cohort Study in Ontario, Canada. *Diabetes care* 2024, 47(9):1568-1575. PMID:38820461. https://doi.org/10.2337/dc24-0108.
2. Kosidou K et al: Maternal Steroid Hormone Levels in Early Pregnancy and Autism in the Offspring: A Population-Based, Nested Case-Control Study. *Biological psychiatry* 2024, 96(2):147-158. PMID:38752911. https://doi.org/10.1016/j.biopsych.2024.02.1014.
3. Jia Q et al: Transcript levels of 4 genes in umbilical cord blood are predictive of later autism development: a longitudinal follow-up study. *Journal of psychiatry & neuroscience : JPN* 2023, 48(5):E334-e344. PMID:37673435. https://doi.org/10.1503/jpn.230046.
4. Cheng W et al: Identification of a robust non-coding RNA signature in diagnosing autism spectrum disorder by cross-validation of microarray data from peripheral blood samples. *Medicine* 2020, 99(11):e19484. PMID:32176083. https://doi.org/10.1097/md.0000000000019484.
5. Mesleh A et al: Blood Proteomics Analysis Reveals Potential Biomarkers and Convergent Dysregulated Pathways in Autism Spectrum Disorder: A Pilot Study. *International journal of molecular sciences* 2023, 24(8). PMID:37108604. https://doi.org/10.3390/ijms24087443.
6. Cao X et al: A Systematic Investigation of Complement and Coagulation-Related Protein in Autism Spectrum Disorder Using Multiple Reaction Monitoring Technology. *Neuroscience bulletin* 2023, 39(11):1623-1637. PMID:37031449. https://doi.org/10.1007/s12264-023-01055-4.
7. Al-Saei A et al: Validation of plasma protein glycation and oxidation biomarkers for the diagnosis of autism. *Molecular psychiatry* 2024, 29(3):653-659. PMID:38135754. https://doi.org/10.1038/s41380-023-02357-9.
8. Shen Y et al: Autism spectrum disorder and severe social impairment associated with elevated plasma interleukin-8. *Pediatric research* 2021, 89(3):591-597. PMID:32330928. https://doi.org/10.1038/s41390-020-0910-x.
9. Damodaran LP et al: Urinary oxidative stress markers in children with autism. *Redox report : communications in free radical research* 2011, 16(5):216-222. PMID:22005342. https://doi.org/10.1179/1351000211y.0000000012.
